# Usability and Acceptability of Electronic Immunization Registry Data Entry Workflows From the Health Care Worker Perspective in Siaya, Kenya (Part 3): Pre-Post Study

**DOI:** 10.2196/39383

**Published:** 2023-03-30

**Authors:** Rachel Wittenauer, Samantha B Dolan, Anne Njoroge, Penina Onyango, George Owiso, Peter Rabinowitz, Nancy Puttkammer

**Affiliations:** 1 Department of Global Health University of Washington Seattle, WA United States; 2 International Training and Education Center for Health University of Washington Seattle, WA United States; 3 County Department of Health Siaya County Siaya Kenya

**Keywords:** workflow, digital health, electronic immunization registry, usability, acceptability, health care worker, technology adoption

## Abstract

**Background:**

Digital health tools such as electronic immunization registries (EIRs) have the potential to improve patient care and alleviate the challenges that arise from the use of paper-based clinic records for reporting. To address some of these challenges, the Kenya Ministry of Health and the International Training and Education Center for Health Kenya implemented an EIR system in 161 immunizing clinics in Siaya County between 2018 and 2019. The successful implementation of digital health tools depends on many factors, one of which is alignment between the technology and the context in which it is used. One important aspect of that implementation context is the perceptions of the health care workers (HCWs) using the EIR.

**Objective:**

This study aimed to evaluate HCWs’ perceptions of the usability and acceptability of multiple clinic workflows using the new EIR.

**Methods:**

We performed a mixed methods pre-post study using semistructured interviews with HCWs at 6 facilities in Siaya County, Kenya. We interviewed HCWs at each facility 4 times: at baseline and once after the implementation of 3 different workflow modifications (n=24 interviews). The baseline state was dual data entry with paper records and the EIR. We then implemented 3 workflow modifications for 1 full day each: fully paperless data entry, preparation of an appointment diary before patient visits for the day, and a combination of the 2 workflows. We compared ratings and themes across interviews after each of the 4 workflows to understand the changes in the usability and acceptability of the EIR.

**Results:**

HCWs considered the EIR clinic workflows to be usable and acceptable. Of the modified workflows, HCWs perceived the fully paperless workflow most favorably. In all workflows, HCWs’ perceived benefits included ease of clinical decision-making using the EIR, reduced mental burden of data entry when using the EIR, and ease of identification of errors. Perceived barriers to the workflow included contextual challenges such as staffing shortages and lack of network connectivity, EIR platform challenges such as errors in saving records and missing fields, and workflow challenges such as the dual data entry burden of using paper and digital tools simultaneously.

**Conclusions:**

Fully paperless EIR implementation shows great promise from a workflow acceptability standpoint, contingent upon the presence of supporting contextual clinic factors and the resolution of system performance and design challenges. Rather than trying to identify a singular best workflow, future efforts should provide adequate flexibility for HCWs to implement the new system in their unique clinic context. Future EIR implementation stands to benefit from continued monitoring of EIR adoption acceptability during implementation, both for Siaya’s program and for other efforts around the globe, as digital health interventions become more widely used.

## Introduction

### Background

Vaccines are one of the safest and most cost-effective public health interventions for reducing morbidity and mortality owing to vaccine-preventable diseases worldwide, and every year, vaccines prevent 2 to 3 million deaths globally [1,2]. Before the disruptions owing to COVID-19, Kenya exceeded the Universal Child Immunization goals of at least 80% of children receiving the third dose of diphtheria-tetanus-pertussis immunization each year since 2007 [3]. However, an estimated 450,000 children remain not fully vaccinated in Kenya [4].

Kenya’s Expanded Program on immunization reporting relies primarily on a paper-based reporting system to manage immunization data collected from health facilities on a monthly basis. Immunization services in Kenya are delivered through a network of >12,000 health facilities and clinics throughout the 47 counties in the country [5]. County governments are responsible for providing health services including reporting information on immunization services and coverage [4]. The reporting process is based on paper-based record keeping across as many as 7 different paper tools at each facility, including the Mother and Child Health Booklets, immunization tally sheets, and Immunization Permanent Register, among others. According to the Kenya Ministry of Health’s comprehensive Multi-Year Plan 2015 to 2020, “the counties face several challenges in their ability to critically analyze the immunization data they report and using this information for decision-making” [4]. Addressing challenges such as these by building capacity of frontline health workers, strengthening infrastructure and logistics, and strengthening monitoring and surveillance systems are key components of several global strategies and national action plans, including the Global Vaccine Action Plan and the Kenya Ministry of Health (MOH) Comprehensive Multi-Year Plan for Immunization [4,6,7].

The use of digital health tools such as electronic immunization registries (EIRs) has shown promising results in increasing vaccination uptake, adherence, and timeliness [6,8,9]. However, health informatics technologies can also be disruptive and have unintended consequences including additional workload, overweighted trust in the technology, routine communication interruption, and medical errors if there is a mismatch between the system and the real-world practices of the health care context in which it is implemented [10,11]. The benefits that EIRs promise can only be realized if they are successfully adopted and integrated into existing patient care processes. Many digital health implementations have failed to be implemented at scale, in part because of incorrect assumptions about the behaviors and preferences of the users of the technology [12,13]. Additional research is needed to understand the benefits and barriers of EIR implementation and what can be done to mitigate barriers including user workload and system usability, which are at the intersection of task and worker elements [14,15].

To mitigate immunization reporting and surveillance challenges stemming from this reliance on multiple paper-based tools, the International Training and Education Center for Health (I-TECH) Kenya, affiliated with the University of Washington, partnered with the Kenya MOH to digitize immunization records in 1 pilot county: Siaya County in western Kenya. I-TECH Kenya and the MOH developed an EIR that can manage electronic vaccine records from multiple health facilities and is based on the OpenSRP and OpenMRS open-source platforms [16,17]. The EIR, called the Kenya Immunization Platform, is a tablet-based platform that allows health care workers (HCWs) to register children, quickly record vaccines administered, identify children who are missing specific vaccinations, and generate aggregate facility reports. The EIR system on this platform was originally created in Zambia based on stakeholder feedback regarding business processes and user requirements. The I-TECH Kenya team then adapted the EIR to Kenya’s specific health care system and immunization schedule to closely reflect the pre-existing paper-based record system. Data elements collected by the EIR system include mother’s contact information, child’s height and weight, immunizations received, insecticide-treated net and vitamin A received, and other fields in the current standard paper-based reporting tools. After the successful pilot phase in 20 facilities of Gem subcounty, the EIR was implemented in all 161 immunizing clinics in Siaya County between 2018 and 2019. This study is part of a comprehensive evaluation of the implementation of an EIR in Siaya County, including a baseline usability study and time-in-motion study [18,19].

### Objectives

The portion of the evaluation reported here assesses point-of-care workflows using the EIR from the perspective of the HCWs who are using the system. The aim of this study was to understand HCWs’ attitudes and perceptions of the usability and acceptability of different clinic workflows using the EIR as part of the time-in-motion study. When redesigning work, implementers should consider different elements of a work system including the individual, tasks, tools and technologies, physical environment, and organizational conditions [20]. By identifying factors that positively and negatively affect the usability and acceptability of the EIR, our research sought to inform strategies to improve EIR adoption and consequently data quality, data demand, and data use in MOH efforts to improve national vaccination coverage [6].

## Methods

This is a pre-post study to evaluate 3 different integrations of the EIR into clinic workflows, as compared with the baseline data entry workflow, with regard to the usability and acceptability of the EIR digital tool.

### Conceptual Framework for Study

Technology adoption in a clinical environment depends on many factors that can vary from system to system and from user to user. One useful framework summarizing these factors is the “Fit between Individuals, Task and Technology” framework, which proposes that technology adoption depends on the fit between the attributes of the individual, attributes of the technology, and attributes of the clinical tasks and processes [21,22]. In the context of our study, because adoption of the EIR was mandatory, we will evaluate the usability and acceptability of the EIR within the clinic workflows as a proxy for “adoption.”

### Study Design

#### Overview

This study applied a mixed methods approach to assess how modifications made to the immunization clinic workflow among facilities using EIRs affected HCWs’ perceptions of usability and acceptability of the EIR based on this framework. A pre-post study was conducted to examine 3 different workflow modifications and their effects on the usability and acceptability of the EIR workflow. Baseline clinic observations and interviews were conducted in the first week of the study. In the second week, modifications to the clinic workflows were implemented, and changes in HCW interview responses through semistructured interviews were evaluated. The interviews had both quantitative measures, described in the Quantitative Analysis Methods section, and open-ended interview questions that solicited information on the ease of use of the EIR application, complexity of the workflow, time pressure, preference among the implemented workflows, and workflow challenges and recommendations. The purpose of this study was exploratory and hypothesis generating.

#### Study Setting and Facility Sampling

There are 161 immunizing facilities in Siaya County, and the EIR was implemented in all of them over a 2-year period. The EIR was first implemented in 20 facilities in Gem subcounty of Siaya County, which was chosen because the program has the most experience working with the MOH and facility staff members in these counties, which simplified the data collection and logistics processes. Sampling was restricted to facilities that had been using the EIR for >3 months to ensure complete adoption of the application by each facility. Among these facilities, 6 public facilities distributed across the subcounty of different sizes (small vs large, based on the size of the facility catchment area) were selected to explore possible differences in acceptability and usability by clinic size. The facility types are summarized in Table 1.

**Table 1:** Facilities selected for workflow modification in Gem subcounty.

Facility	Facility type	Facility size
Facility A	Level 2: dispensary	Small facility
Facility B	Level 2: dispensary	Small facility
Facility C	Level 3: health center	Large facility
Facility D	Level 3: health center	Large facility
Facility E	Level 3: health center	Large facility
Facility F	Level 4: county referral hospital	Large facility

Each selected facility had 1 tablet with the EIR application installed on it. Some facilities had only 1 HCW using the EIR, whereas others had multiple HCWs using the tablet. In all facilities, record keeping was performed using both the EIR and paper-based records simultaneously. At each facility, interview informants were selected based on which HCW was on duty and using the EIR during the 2-week data collection period. One HCW from each of the 6 facilities was interviewed, and each HCW conducted 4 interviews: one at baseline and one after each workflow modification was implemented. Thus, we conducted 24 interviews representing the usability and acceptability of 4 workflows from the perspective of 6 HCWs representing 6 different health facilities. The HCWs interviewed were typically nurses (15/24, 63%), had been using the EIR for >3 months (13/24, 54%), and had been working at their health care facility for between 1 and 5 years (18/24, 75%).

#### Intervention: Workflow Modifications to Optimize EIR Implementation

The 3 specific interventions chosen and the rationale behind piloting them are described in Table 2.

Each workflow was implemented for a 1-day period during which a minimum of 5 immunization visits were observed. The “preparation” workflow was implemented on the first day, the “paperless” workflow was implemented on the second day, and the “preparation” and “paperless” workflow modifications were both implemented on the third day to understand their joint benefits and challenges. The implementation of the workflows consisted of training the HCW at the beginning of the day, wherein the data collector reviewed the workflow modification with the HCW. Immunization visits and observations took place as they did at baseline, except for the modified workflow in place. The same steps to implement the modified workflows occurred at all 6 facilities, with 1 data collector assigned to each facility for the week and working with the same HCW.

**Table 2:** Workflow modifications and rationale.

Workflow	Description of workflow modification	Anticipated benefits
Baseline	The existing workflow in the immunization clinics consisted of dual data entry in both paper registers and the EIR^a^ concurrently during each immunization session. Upon initial introduction of the EIR, facilities were not instructed to adopt any workflow specifically, but rather each facility integrated the EIR into their clinic activities organically.	Not applicable.
Preparation	Before the immunization clinic started seeing children in the morning, the HCW^b^ prepared the list of expected appointments for the day using the paper registers. Then, the HCW confirmed that each expected child is entered in the EIR and their information is up to date. The immunization clinic visits then proceeded as normal.	Inconsistencies between paper-based and EIR records were identified before the session and completed in a batch for all expected children rather than being identified and corrected while the mother-child pair was present and waiting. We expected this to save time and thereby improve the usability and acceptability of the EIR.
Paperless	HCWs removed the steps involving paper records during the immunization visit sessions. Instead, only the EIR was used during the session.	By removing the dual data entry aspect of the baseline workflow, we expected that use of the EIR will be perceived as being less complicated, involving less time pressure, and broadly be more acceptable and usable as defined by our guiding framework.
Preparation plus paperless	HCWs implement the preparation and paperless workflows on the same day. That is, appointment lists were prepared in the morning, and the clinic workflow proceeded without the use of paper records.	Benefits, challenges, and interactions of the 2 workflows were jointly assessed.

^a^EIR: electronic immunization registry.

^b^HCW: health care worker.

#### Measures of Interest

In this study, the EIR was implemented throughout all facilities using a technology-push approach [23]. As “adoption” of the technology being implemented was mandatory, rather than evaluating the degree of technology adoption, instead the success of that adoption was evaluated by adapting our outcome of interest to be “usability and acceptability” of the EIR’s integration into different clinic workflows. A workflow is the “sequence of physical and mental tasks performed by various people within and between work environments,” and it can occur sequentially or simultaneously as well as at several levels from within a single person to across entire organizations [24]. The specific workflow assessed in this study was the sequence of data entry actions performed by a HCW within an immunization clinic.

Usability and acceptability have been defined as distinct concepts. Sekhon et al [25] defined acceptability as a construct reflecting the extent to which people perceive a health care intervention to be appropriate. Usability is defined by the US Food and Drug Administration as the characteristics of the user interface of the product, which establish effectiveness, efficiency, and user satisfaction [26,27]. Due to this study’s emphasis on the clinic workflow and not the EIR technology in isolation, in this evaluation, we evaluated HCWs’ perception of “usability and acceptability” as a joint construct. The clinic workflow with the EIR inherently depends on both the ease of use of the digital platform and the integration of the technology within clinic activities; thus, the perceived benefits and challenges of the workflow are not classifiable as related only to usability or acceptability.

Interview questions from previously validated tools regarding system use and user satisfaction were adapted for this study:

- *The National Aeronautics and Space Administration (NASA) Task Load Index measures* ask users to describe functions according to the amount of “time pressure” they face, frustration experienced, mental demand needed, effort involved, and confidence in their performance [28].
- *Likert scale–based workflow acceptability measures* ask users to rate on a scale of “Strongly Disagree” to “Strongly Agree” their opinions on questions including “I have enough time to vaccinate all patients attending an immunization clinic,” “The clinic workflow is too complicated,” “We have enough tablets for our clinic to use [the EIR],” “We have enough staff to adequately use [the EIR] during our immunization clinic,” and “I find [the EIR] easy to use.”

#### Data Collection

Data were collected using semistructured interviews lasting approximately 30 minutes each. Data collectors recorded responses to open-ended questions by taking notes on the paper-based data collection tools during the interviews. All the interviews were conducted in English.

To successfully complete all interviews within time and budget constraints of the evaluation, 4 data collectors were trained to assist with the interviews during the 2-week data collection period. Data collectors were hired in the same county where the study took place, and all data collectors had past experience collecting data for other health facility–based research studies. The data collector training took place over the course of 2 days and included a review of the purpose of the study, mock interviews with our data collection group, and a pilot session of the data collection materials at a nearby clinic.

#### Ethics Approval, Informed Consent, and Participation

This study was determined to be a nonhuman subjects research by the University of Washington Institutional Review Board (STUDY00006256) and received human subjects’ ethics approval from Amref Kenya (ESRC P587-2019), as a routine program evaluation.

The research team obtained informed consent from all the HCWs observed. Consent was documented for each HCW via a written form in English after reading a summary of the goals of the immunization observations (Multimedia Appendix 1). All names of the HCWs and health facilities were anonymized to protect participants’ confidentiality. The HCWs participating in this study were not compensated for this research.

### Data Analysis

#### Qualitative Analysis Methods

We used a thematic coding approach to code the qualitative data collected using the interview tools. The 2 coders (RW and SD) were the 2 primary data collectors and were English speakers affiliated with the program that implemented the EIR. Codebook development began deductively by defining high-level themes related to the guiding frameworks for adoption, usability, and acceptability. The 2 primary data collectors then transcribed a pilot set of 2 baseline and modified workflow interview transcripts with an inductive perspective to account for emerging codes that were not included in the prescribed frameworks. The coders then convened and reviewed all the applied codes and revised the codebook definitions accordingly, including the addition of new codes that had surfaced. The 2 data collectors then coded all the interviews using the updated codes. Finally, the coders compared every code and discussed discrepancies until all codes matched. The coded transcripts were used to assess and group findings on usability and acceptability using the clinic workflow. These activities were performed using Microsoft Excel and the ATLAS.ti software.

#### Quantitative Analysis Methods

The quantitative analysis focused on HCWs’ responses to the NASA Task Load Index and Likert scale–based measures. To understand the changes by each workflow, median scores for each scale and workflow type were calculated; however, statistical methods to evaluate differences between each modified workflow and baseline were not applied owing to inadequate sample sizes.

To understand changes in EIR workflow usability and acceptability at the level of the individual facility, the direction of the score change (improved, worsened, and no change compared with baseline) was evaluated by comparing the facility-specific score for each indicator in the modified workflow versus baseline. A composite indicator for each measure was calculated by summing the ratings given by each HCW and evaluating whether this total score was higher or lower at baseline versus in each modified workflow. All calculations were performed using R Studio software (R Core Team 2022).

## Results

### Findings From Quantitative Survey Responses

Usability and acceptability improved in modified workflows compared with baseline, as indicated by a higher median Task Load Index score across health facilities (Table 3). For instance, the median score for mental demand was 6 at baseline and improved to median scores of 8, 9, and 9 in the preparation, paperless, and combined workflow, respectively. A similar trend was observed for the 3 dimensions of time pressure, effort, and frustration, whereas performance was rated similarly in all 4 workflows.

**Table 3:** Median National Aeronautics and Space Administration (NASA) Task Load Index scores by workflow (N=6).

Indicator for each facility	Baseline	Preparation	Paperless	Preparation plus paperless
	Rating, median (range)^a^	Rating, median (range)^a^	Rating, median (range)^a^	Rating, median (range)^a^
Mental demand	6 (1-10)	8 (4-10)^b^	9 (8-10)	9 (7-9)^b^
Time pressure	5 (3-10)	8 (3-9)^b^	10 (8-10)	9 (8-10)^b^
Your performance	8 (8-10)	8 (6-9)^b^	9 (9-10)^b^	8 (8-10)^b^
Effort	6.5 (2-10)	8 (5-9)^b^	9 (9-10)	8 (8-10)^b^
Frustration	7 (2-8)	9 (3-10)^b^	9 (6-10)	8 (6-10)^b^

^a^NASA Task Load Index dimensions were measured on a scale of 1 to 10, with 10 being the most favorable score and 1 being the least favorable score.

^b^n=5 for these scores; 1 facility had missing data owing to electronic immunization registry malfunction during the observation day.

HCWs’ responses to the Likert scale–based acceptability indicators showed mixed results (Table 4). For instance, HCWs’ ratings in response to the question “I have enough time to vaccinate all patients attending an immunization clinic” were a median of 4 (“Agree” on the Likert scale) at baseline and a median of 4, 5, and 4 in the preparation, paperless, and combined workflows, respectively.

These measures did not reveal any differences in the usability and acceptability of the EIR within small versus large facilities. The change in usability and acceptability scores by indicator at each participating facility can be found in Multimedia Appendix 2.

**Table 4:** Median Likert scale scores by workflow (N=6).

Indicator for each facility	Baseline	Preparation	Paperless	Preparation plus paperless
	Rating, median (range)^a^	Rating, median (range)^a^	Rating, median (range)^a^	Rating, median (range)^a^
Enough time to vaccinate patients	4 (4-5)	4 (4-5)^b^	5 (4-5)	4 (4-4)^b^
Workflow not too complex	—^c^	4 (4-5)^b^	4.5 (4-5)	4 (4-5)^b^
Enough EIR^d^ tablets for workflow	4 (2-5)	4 (2-5)^b^	4 (1-5)	4 (4-5)^b^
Enough staff members for workflow	3 (2-5)	4 (2-4)^b^	3 (1-5)	4 (2-5)^b^
EIR easy to use	5 (4-5)	4 (4-5)^b^	4.5 (4-5)	4 (4-5)^b^

^a^Factors were measured on a Likert scale of 1 to 5, with 5 being the most favorable score and 1 being the least favorable score.

^b^n=5 for these scores; 1 facility had missing data owing to EIR malfunction during the observation day.

^c^The question was not asked in a consistent format between the baseline and modified workflow interviews, preventing comparison between baseline and each of the modifications.

^d^EIR: electronic immunization registry.

### Findings From Qualitative Interview Responses

#### Overview

The findings from the key informant interviews support the scale-based ratings in some ways and contradict or offer caveats to the ratings in other aspects (Table 5).

**Table 5:** Summary of qualitative findings on workflow usability and acceptability.

Workflow	Key workflow benefits	Key workflow challenges
Baseline	The EIR^a^ platform was broadly well liked by HCWs^b^ The EIR platform eased certain clinical decision-making and administrative tasks during patient visits	The time-intensive effort of searching for and entering the same data in multiple places at once EIR application challenges such as frequent freezing and no network connection to synchronize records with the server Errors in the EIR application itself, which take time attempting to resolve with the patient in the room
Preparation	Some facilities indicated that once the list was created, it saved time during clinic visits The process of creating the list facilitated identification of EIR errors before the patient visit	Creation of the appointment list from solely the permanent register and daily activity register was time consuming HCWs were more likely to perceive the preparation process as a waste of time if the parents and children did not show up for their expected appointments
Paperless	HCWs widely perceive the workflow to save time because of not having to enter data twice, be less mentally taxing because the application calculates and prompts action step-by-step, and prevent errors by displaying all the information needed immediately at the point of care	When the EIR was not working, the workflow caused frustration, confusion, and potential errors
Preparation plus paperless	User perceptions of the preparation plus paperless combined workflow modification did not have meaningful differences compared with either workflow implemented separately	NR^c^

^a^EIR: electronic immunization registry.

^b^HCWs: health care workers.

^c^NR: not reported. Results from this workflow were the same as reported for the other workflows.

#### Baseline Workflow

In general, the HCWs who used the platform described the EIR favorably. They perceived it to be easy to navigate, prevent errors, ease reporting, and that it was a desired future tool to house immunization records. Some clinics have also noted the portability of the system to be an advantage. At 1 clinic where the maternal ward and immunization clinic were physically separated, the HCW was able to carry the EIR tablet to administer an immunization to a child that was just born. The HCW commented that this was preferable for both themselves and the new mother, as opposed to before when either the HCW had to bring multiple large paper registers to the clinic or the mother had to bring the new child physically to the immunization room to receive and record the immunizations. In this case, the EIR provided the HCW with the flexibility to adapt data collection to be a more appropriate workflow for their physical clinic context.

However, there are pervasive challenges with how the EIR integrates into the workflow in its current form, both because of the technology itself and contextual factors relating to the clinic environment. Three of the most common EIR platform–specific challenges described by HCWs in the baseline workflow were regarding duplicate data entry, EIR performance, and EIR application design, described as follows:

- *Duplicate data entry*: the time-intensive effort to search for and enter the same data in multiple places simultaneously during this workflow, notably the permanent register, daily activity register, mother-child booklet, and the EIR itself
- *EIR performance and network reliability*: obstacles related to the EIR tablet such as frequent freezing, no network connection to synchronize records with the server, and the disappearance of saved records necessitating reregistration of the children
- *EIR application design*: errors in the EIR application itself that are inconsistent with clinical practice such as missing antigens in the application and error messages incorrectly indicating default on immunizations

In addition to EIR-specific challenges (which were not remedied for the duration of the data collection period), the baseline interviews highlighted several additional factors that also affected the findings in the subsequent workflow modifications. Some of these factors were that every facility had a unique physical layout, different staff member roles at each step of the patient flow, varying numbers of patients, degree of internet connectivity, and other contextual variables that affected the workflow. For example, several clinics conducted growth monitoring before all immunization visits at a separate desk in the facility, whereas others conducted all activities in the room where immunizations were delivered. These physical differences and associated staff member positions affected the flow of data entry. In addition, several HCWs remarked that they were never trained formally and learned how to use the EIR while on the job, which some saw as a weakness, and others did not consider it to be a challenge. Another example was in clinics that had to repurpose other staff members to enter data in one tool (either paper or the EIR) while the HCW entered data in the other. In these instances, the workload required of the HCWs by the implementation of the new EIR exacerbated the existing constraints, and the dual data entry caused frustration and additional time pressure felt by the HCW. There were no differences in the perceived challenges or benefits between small and large facilities.

#### Preparation Modified Workflow

HCWs were divided based on the success of the “preparation” workflow. Their biggest concern with the preparation workflow was the amount of time it took to create the appointment list; however, several facilities reported that once the list was made, it saved time and helped identify errors early. We observed that facilities implemented the “preparation” workflow in 2 different ways, despite the same instructions being given. In 2 of the facilities, HCWs created an appointment diary by collecting the mother-child booklets of the patients who had already arrived in the facility that morning and were in the waiting area, checking their records in the EIR for accuracy or registering the child if there was no EIR ID recorded, and then returning the booklets back to the mothers. In the other facilities, the HCW created the appointment diary using the 2 paper registers to cross-check for expected children before any patients arrived, which provided details on which children were now defaulting on their immunizations, though it took substantially more time to create.

Some HCWs perceived some positive effects of the workflow, such as the patient workflow being less mentally demanding once the list was made and easing time pressure during visits. Several facilities reported that once the list was made, it saved time and helped identify errors early—1 facility identified an error in the patient EIR record during the creation of the list and corrected it, which saved the HCW from having to do that task with a patient waiting in the room. Interestingly, 1 HCW had already been creating a similar appointment diary every evening and calling the mothers with appointments to remind them to come in the next day. This meant the “intervention” workflow was the same as her usual routine, so even though it was perceived as more demanding by some HCWs, for this individual, it was the same as the baseline workflow and considered usable and acceptable.

However, several HCWs also reported that creating the list took too long and was not a good use of time. This was particularly true in instances where either patients were due for a visit that day and did not show up for their expected appointment or patients arrived without an appointment. In 1 large health facility, 23 appointments were expected that day, but only 9 children came to the clinic for vaccinations.

The reported challenges and benefits of the preparation workflow did not vary according to the facility size. One small facility had a favorable perception of the workflow, whereas the other had a negative perception. Perceptions of usability and acceptability in larger facilities varied similarly.

#### Paperless Modified Workflow

Every HCW interviewed preferred the paperless workflow to the baseline (dual data entry) workflow, with the important caveat that this preference was tied to whether the EIR was functional and not missing records. Participants widely perceived the paperless workflow to save time because they did not have to enter data twice, be less mentally taxing because the application calculates and prompts action step-by-step, and prevent errors by displaying all the information needed immediately at the point of care.

However, we only observed these benefits when the EIR application was functioning fully as designed, namely, when the network was working, records were synchronized with the server, and there were no errors in saving the vaccines that were administered in the record. When the EIR was not working, the workflow caused frustration and confusion. One HCW also noted with frustration one day that when the EIR was not working, she still had to administer immunizations, but she often updated the EIR record based on memory, which the HCW felt was unreliable and prone to errors. For 1 facility, we could not collect data on paperless workflows because the EIR system was not operational.

During this workflow implementation, several HCWs also noted that, based on the flow of patients in the immunization clinic, it would be more helpful for the EIR to include growth monitoring capabilities that align directly with the longitudinal capabilities currently logged in the mother-child booklets. In many of the clinics, the physical layout of activities had initial information intake for mothers and children occur first at a station to measure and record growth monitoring, before entering the immunization clinic itself. However, the EIR did not include a module to record growth monitoring information longitudinally in the same way; therefore, this order of clinic operations was not consistent with the EIR technology in most observed facilities.

All HCWs expressed a desire to move to a paperless data entry workflow once these major issues with platform functionality were addressed. The usability and acceptability of the workflow did not vary according to the facility size.

#### Preparation Plus Paperless Combined Modified Workflow

On the basis of interview conversations, user perceptions of the *preparation* plus *paperless* combined workflow modification did not have meaningful differences compared with either workflow implemented separately. For example, issues with EIR functionality remained common complaints with the workflow, as they did in the paperless-only workflow of the previous day. Similar challenges to the preparation workflow such as the perceived waste of time and time-intensive processes to create the appointment diary were also noticed. However, 1 HCW stated that they preferred this combined workflow out of the 3 options. As with the “preparation” and “paperless” workflows separately, there were no differences between small and large facilities in the perceptions of usability and acceptability of this combined workflow.

## Discussion

### Summary

Several themes emerged from the interviews before and after the 3 workflow modifications. At a high level, participants indicated almost universally that the paperless workflow was more acceptable than the baseline dual data entry workflow, whereas the dual data entry, preparation, and preparation plus paperless workflows had more mixed receptions. There were several benefits perceived by the HCWs in modifying the workflows for the use of a paperless process and preparation of a daily appointment list. However, there were also several challenges that must be addressed in the clinic environment, such as the number of staff members and network connectivity, and the EIR platform itself, such as freezing and missing records, before these new workflows are considered for broader implementation.

### Principal Findings

#### Overview

These findings can be explained by the several layering elements of the clinic context, EIR system, and workflow, which are all interrelated [29] and can have cascading effects, positive or negative, on the ultimate usability and acceptability of EIR implementation from the HCW perspective [21,29,30]. The EIR application itself is the same at each facility; however, the way it is integrated into the workflow can take many different forms. These results demonstrate that when HCWs are given (1) an adequate enabling environment; (2) a reliable EIR system; and (3) flexibility in the workflow, the acceptability of the workflow may be enhanced, ultimately leading to improved data quality and use [6]. These findings add to the growing body of literature on the upscaling of EIRs and other digital health technologies.

Notably, this study was conducted in 2019 before the COVID-19 pandemic, although the EIR application is still in use in the pilot county. As the MOH efforts eventually shift from COVID-19 to addressing other challenges such as routine childhood immunizations, this EIR application is being considered for scale-up in other areas of the country.

#### Enabling Clinic Environment

Contextual challenges with technology, personnel, and enabling environment levels can be barriers to smooth EIR workflows [15,20,29]. Each clinic had a unique context, from the configuration of individual staff members to the clinic flow of activities to the environmental elements. This context can either precondition that the EIR is successfully integrated into the workflow or have negative consequences for the acceptability of the EIR. The lack of network connectivity, in particular, was a common barrier both in our results and in other EIR evaluations [15]. These workflow modifications, notably the fully paperless workflow, were able to improve the way HCWs interact with the EIR during immunization sessions but could not change the surrounding clinic context. This was seen, for example, in the quantitative indicators that had to do with staffing and number of tablets (context based) whose values did not change, compared with the indicators that asked questions solely about how the HCW and the system interacted (eg, mental demand, frustration), which changed upon modifying the workflows.

#### Reliable EIR System

Even if a clinic’s context is well suited for EIR adoption, if the system is poorly designed without the HCW needs in mind, then it will have cascading negative effects on acceptability from the HCW [12,31,32]. These results show that without system reliability and well-designed software, HCWs were unable to use the EIR effectively in their workplace, leading to lower acceptability and possibly data quality. Reliable and well-designed digital health technologies have documented positive effects on system acceptability [9,15]. However, even if a technology operates perfectly as designed, it can still have negative consequences depending on how it is used [11]. Rather than focusing on the technology itself, this study emphasizes the acceptability flow of work surrounding the system. In future EIR implementations, it will be crucial to collaborate with HCWs to solicit input and conduct pilot testing periods at length to identify and meet system requirements.

#### Workflow and System Match

Without the workflow and system meeting the real-world needs of HCWs, the EIR data entry workflow was not considered acceptable by the HCWs. These results revealed many examples of consistent and inconsistent matches between clinic reality and system design in the workflows we evaluated, such as HCWs being able to carry the portable EIR tablet to the maternity ward (a good match) and some clinics repurposing staff members for dual data entry (lack of match between workflow and system). In addition to these EIR design aspects, instances of errors in patient data entry resulting from a workflow mismatch were observed. For example, when the clinic was too busy to allow for dual data entry, HCWs reported that they were obligated to fill in records based on memory, which made the records more prone to errors. Thus, some EIR data entry errors were rooted both in the technology itself and in the workflow surrounding that technology. As described by the Institute of Medicine in their landmark 1999 report on medical errors, “all technology introduces new errors, even when its sole purpose is to prevent errors” [33]. Organizations implementing new technologies, such as EIRs, should anticipate this and seek strategies to mitigate these errors whenever possible [11].

These findings suggest that one of these strategies is to empower HCWs with the flexibility to use the new EIR in a manner that is appropriate for their context and pre-existing operations. This is supported by findings from previous studies on the parallel development of both technical systems and the social systems that surround them [30,34]. These studies indicated that systems should be designed with “minimum critical specification” such that users can develop appropriate configurations to suit their needs. To further enable facility-level adoption and integration of the EIR into the workflow, HCW-led support measures such as peer-to-peer support groups, champion-based approaches, and other user-driven mechanisms could potentially be valuable means of identifying best practices for implementation in specific contexts, although additional research is needed on their utility in different contexts.

### Limitations and Strengths

This evaluation provides a valuable exploration of the challenges and benefits of EIR workflow implementation from the perspective of HCWs. First, much of the existing evidence on the adoption of EIRs and other digital health technologies has focused on application development or the before and after effects of implementation on immunization service coverage [15]. These findings contribute to filling this gap by understanding the factors that facilitate the adoption of digital health technologies. Second, the qualitative component of this mixed methods study design facilitated important insights into the match between the EIR system and the clinic context where it is used, which were not revealed by quantitative measures alone. Third, this approach of short-duration intervention simulations provided evidence quickly and helped fill information gaps rapidly in an implementation context [35].

This study has several limitations. First, in implementing the workflow modifications, it is possible that a single day of training to orient the HCW to the new workflow and conduct data collection was not a long enough period for it to be adopted and measured as intended. It is possible that the observations and interviews with HCWs were influenced by the Hawthorne effect. Second, the sample size also did not allow for statistical assessment of effect modifiers such as facility size, which could be addressed in future studies evaluating a larger set of facilities. However, although 6 individuals is a small sample size for most statistical tests, it is generally considered acceptable for usability testing and heuristic evaluation [36]. Finally, the quantitative scales we used have not been validated in other clinic settings, limiting our ability to generalize to other contexts or draw conclusions about the relationship between the quantitative scores and the future likelihood of the acceptability of the EIR. This study did not seek to systematically evaluate a range of clinic context factors as present or absent, although future studies in a wider set of sites could undertake this type of work to assess a variety of contextual conditions and examine whether workflow modifications work better in some contexts but not others.

### Conclusions

Fully paperless EIR implementation shows great promise from a workflow acceptability standpoint, contingent upon the presence of supporting contextual factors and the resolution of system performance and design challenges. A reliable EIR will not be designed with a one-size-fits-all approach and will instead provide adequate flexibility for HCWs to implement the new system in their unique clinic context. Future studies in this area, rather than trying to identify a singular best workflow, should look for ways to maximize flexibility, reliability of the app, and strong enabling environments. To further enable facility-level adoption and integration of the EIR into the workflow, HCW-led support measures such as peer-to-peer support groups, champion-based approaches, and other user-driven mechanisms could potentially be valuable means of identifying the best practices for implementation in specific contexts. Future EIR implementation stands to benefit from continued monitoring of EIR adoption acceptability during implementation, both for Siaya’s program and for other efforts around the globe, as digital health interventions become more widely used.

## Appendix

Multimedia Appendix 1 Study volunteer consent form.

Multimedia Appendix 2 Data tables.

## Abbreviations

EIR: electronic immunization registry
HCW: health care worker
I-TECH: International Training and Education Center for Health
MOH: Ministry of Health
NASA: National Aeronautics and Space Administration
