# Supplementary material for: Usability and Acceptability of Electronic Immunization Registry Data Entry Workflows From the Health Care Worker Perspective in Siaya, Kenya (Part 3): Pre-Post Study
Source: JMIR Form Res. 2023 Mar 30;7:e39383. doi: 10.2196/39383 (PMC10131729; doi:10.2196/39383)
Supplement: Multimedia Appendix 1 [file formative_v7i1e39383_app1.docx]

## Appendix 1: Study Consent Form

**UNIVERSITY OF WASHINGTON / I-TECH KENYA**

Global Health Security Partnership Engagement- Expanding Efforts and Strategies to Protect and Improve Public Health Globally through strengthening immunization, surveillance, and laboratory information systems

**Researchers’ Statement**

We are asking you to be in an evaluation because you are a healthcare worker or an affiliated staff member in a health facility where training is being implemented for the Kenya Immunization Platform (KIP). We feel that your experiences with implementing and using KIP will help us create a better health services for health facilities in the future. The purpose of this consent form is to give you the information you will need to help you decide whether to be in our evaluation or not. Please read the form carefully. You may ask questions about the purpose of the evaluation, what we would ask you to do, the possible risks and benefits, your rights as a volunteer, and anything else about the evaluation or this form that is not clear. When we have answered all your questions, you can decide if you want to be in the evaluation or not. We will give you a copy of this form for your records.

**Purpose of evaluation project**

The Kenya Ministry of Health and the University of Washington are doing a programmatic evaluation on best practices for introducing the Kenya Immunization Platform program to health facilities in Kenya. We are giving you this information because we would like you to participate in our evaluation project. If you prefer not to participate, you are free to choose to do so. You will continue to carry out your normal professional duties, with no negative impact. Members of our team are here to help you understand more about the project.

**Evaluation procedures**

- You may be invited to participate in a survey that seeks to get your feedback on KIP’s interface, functionality, and reliability, along with any other opinions you may have about the system.
- You may be invited to be interviewed on your experience using KIP, your opinions of the system, and your suggestions for future improvements needed to make the system better. A researcher will take written notes of your discussion.
- You may be asked about the barriers to implementing activities in your hospital, how health workers can comply with recommended activities, how training and communication can be improved, and training needs among health care workers for effectively practicing activities.
- If changes are made to the evaluation or new information becomes available, you will be informed.

This evaluation site visits will take place over a 1-year period. You may be asked to participate in up to three surveys or interviews. Each group survey or interview will be scheduled around your availability and will take no longer than 1 hour.

**Benefits of the evaluation**

There is no direct benefit to you for participating in this evaluation. However, your participation is likely to help us find out more about how to improve immunization practices among health facilities in Kenya. You will be able to provide opinions that could help to improve healthcare practices in Kenya.

**Risks or discomfort**

There is a risk that you may feel uncomfortable talking about some of the topics in this evaluation. However, we do not wish for this to happen. You do not have to answer any question or take part in a survey or interview if you feel the questions are difficult or if talking about them makes you uncomfortable. You may refuse to participate and you are free to withdraw from this evaluation at any time.

**Alternatives to taking part in the study**

You do not have to take part in this evaluation. Your participation is voluntary. Choosing to take part or not take part in this evaluation will not affect your job. If you choose not to take part, you can continue to carry out your professional duties and nothing will change.

**Confidentiality of information**

Following the survey or interview, the research team will keep all information about you and your responses private. An independent document connecting your name and other identifiers to a study ID number will be kept in a password protected computer file, separate from your data. This document will be destroyed one year after the end of the study.

The knowledge that we get from this evaluation will be shared with you before it is made widely available to the public. No personal names will be used within the reports we produce as a result of this evaluation. The MOH leadership and program managers will receive a written summary of the results. If possible, the research team will meet with you and your clinic staff to discuss the results. Following the meetings, we will publish the results so that other interested people may learn from the evaluation.

**Research-related inquires**

This project has been approved by Amref Kenya ESRC, reference number: AMREF-ESRC P587-2019

If you have any questions related to this research, you may contact George Owiso at I-TECH Kenya ([+254 20 2609340](tel:%2B254%2020%202609340), gowiso@itech-kenya.org) or Dr. Peter Rabinowitz at University of Washington (peterr7@uw.edu ).

If you have questions about your rights as a research participant, you may contact:

The Research Officer

Amref Health Africa in Kenya

Wilson Airport, Lang’ata Road

Office Tel:  +254 20 6994000

Fax: +254 20 606340

P.O Box 30125-00100

Nairobi, Kenya

Your signature on this form means:

- You have been informed about this project’s purpose, procedures, and possible benefits and risks of the evaluation.
- You have been given the chance to ask questions before you sign.
- You have voluntarily agreed to be in this project.

Signed**…………………………………**  Date**……………………………………………**

(Participant)

Signed………………………………… Date…………………………………………………

(Project staff member)
