# Supplementary material for: Usability and Acceptability of Electronic Immunization Registry Data Entry Workflows From the Health Care Worker Perspective in Siaya, Kenya (Part 3): Pre-Post Study
Source: JMIR Form Res. 2023 Mar 30;7:e39383. doi: 10.2196/39383 (PMC10131729; doi:10.2196/39383)
Supplement: Multimedia Appendix 2 [file formative_v7i1e39383_app2.docx]

## Appendix 2: Supplemental Data Tables

Figure 1 illustrates change in usability and acceptability scores by indicator at each facility according to the NASA Task Load Index measures. Based on the composite score, we can see that for the Preparation workflow, 2 out of 5 facilities experienced a net improvement while 3 out of five gave a worse overall rating. For the Paperless workflow, all 6 facilities experienced a net improvement in scores. For the combined Preparation Plus Paperless workflow, 4 facilities experienced a net improvement, 1 worsened, and 1 had no data available.

Figure 1: Facility-level changes in usability and acceptability in each workflow, as measured by the five NASA Task Load Index dimensions


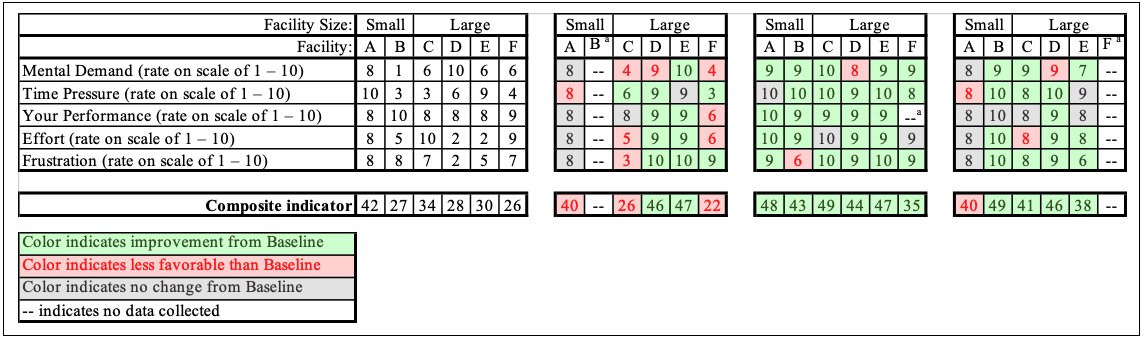


^a^ Data was not collected for these facilities due to platform malfunction during these study-days

Note: A score of 10 is the easiest (better score) and score of 1 is the hardest (lowest score)

Figure 2 illustrates change in usability and acceptability scores by indicator at each facility according to the Likert scale measures. Based on the composite indicator we observed that in the Preparation workflow, 3 out of 5 facilities experienced improvement as compared to their baseline ratings, 1 worsened, and 1 had no change. In the Paperless workflow, 3 out of 6 facilities experienced improvement, 1 worsened, and 2 had no change. In the combined Preparation workflow, zero facilities experienced improvement, 2 worsened, and 3 had no change.

Figure 2: Facility-level changes in usability and acceptability in each workflow, as measured by Likert scale questions


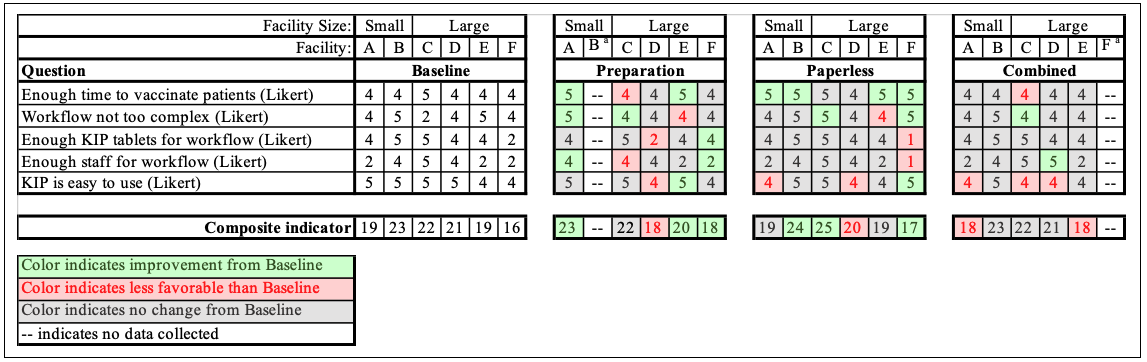


^a^ Data was not collected for these facilities due to platform malfunction during these study days

Note: Score of 1 is least and score of 5 is most favorable
